# A Large Lizard in a Small Islet: Abundance, Body Growth, and Diet of *Podarcis pityusensis* from Es Vaixell (Balearic Islands, Spain)

**DOI:** 10.3390/ani16091314

**Published:** 2026-04-24

**Authors:** Valentín Pérez-Mellado, Ana Pérez-Cembranos

**Affiliations:** Department of Animal Biology, Universidad de Salamanca, 37007 Salamanca, Spain; anapercem@usal.es

**Keywords:** Bledas Islands, Ibiza, Balearics, Squamata, Lacertidae, sex ratio, density

## Abstract

The islet of Vaixell, off the west coast of Ibiza (Balearic Islands, Spain), is home to a native population of the Pityusic wall lizard, *Podarcis pityusensis*, with the largest body size recorded for the species. These lizards live in extreme environmental conditions on an islet with a small surface area covered by very sparse vegetation. The sex ratio is balanced, and a very high incidence of missing toes and autotomized tails is observed, indicating strong intraspecific competition involving both males and females. The body growth rate, adjusted using the Gompertz model, is intense and, apparently, juvenile lizards quickly reach relatively large body sizes. This fast body growth is probably a strategy against predation pressure from conspecifics. In *P. pityusensis* from Vaixell, the peak growth acceleration is prenatal and practically coincides with the moment of hatching. The diet consists mainly of aggregated prey, such as ants, with the inclusion of marine subsidies, such as halophyllous and littoral isopods, and a lower consumption of plant matter compared to other insular populations of lizards from the Balearic Islands. The lizards of Vaixell are an excellent example of the adaptive response of a lacertid lizard to the extreme conditions on the small coastal islets of the Mediterranean, with very small available areas, high population density, but a small population size, of about 50 to 100 lizards, which also reach a remarkable longevity.

## Simple Summary

A native population of the Pityusic wall lizard, *Podarcis pityusensis*, lives on Vaixell Islet (Ibiza, Spain). This population was not studied until 2010, the year of its discovery. Since then, nine visits have allowed us to estimate its population density, the body growth of lizards, the intraspecific competition, and the diet. Lizards grow very rapidly, but their growth appears to slow down before or just after hatching. At this stage, newborn lizards have body sizes that help them escape cannibalism by adults. Although plant cover and food resources are scarce, the population of lizards can reach up to 100 individuals, which survive by exploiting the poor trophic resources, even including marine subsidies such as small crustacean isopods in their diet.

## 1. Introduction

Islands are natural experiment locations that allow us to test theoretical predictions regarding the evolution of natural history traits, such as body size, sexual dimorphism, sex ratio, body growth rates, abundance, or patterns of survival and reproductive effort [1,2]. Small and isolated islands are characterised by a depressed number of terrestrial vertebrates, leading to increased population densities, that is, the so-called density compensation [3]. On several small islets of the Balearic Islands (Spain), there is only one terrestrial vertebrate, an endemic lacertid lizard, the Lilford’s wall lizard, *Podarcis lilfordi* (Günther, 1874) on the Mallorca and Menorca Islands, or the Pityusic wall lizard, *Podarcis pityusensis* (Boscà, 1883) on the Ibiza Islands [4]. This presents an excellent opportunity to test the effect of the lack of terrestrial competitors and predators on the reduction in ecological constraints, the rise in population densities, and the influence on natural history traits [5,6].

Extremely variable densities have been observed in *Podarcis* species inhabiting the Mediterranean islands, ranging from low densities of 100 to 300 individuals per hectare in several populations of Eastern Mediterranean [7] to several hundred or thousand individuals per hectare [6,8,9,10]. In the western Mediterranean, densities on small islets can be very high, both for the Lilford’s wall lizard [10] and the Pityusic wall lizard (unpublished result), although we still lack published data with densities estimated by means of reliable quantitative methods for this species. In addition, it is well known that large body sizes of lizards are common on islands without terrestrial predators [11,12]. Intraspecifically, insular lizards tend to grow to more extreme sizes than mainland lizards [13].

*P. pityusensis* is a robust lizard with an elevated head and rounded snout. The snout–vent length (SVL) can reach almost 100 mm. The coloration and pattern are extremely variable, and a clear sexual dimorphism is observed, both in body size, larger in males, and in coloration, with duller and more cryptic tones in females. The Pityusic wall lizard was studied from a systematic viewpoint during the whole 20th century [14,15,16,17] and references therein. During this research, all known populations were extensively collected and studied, with the description of several subspecies (for a summary, see [4]), as a recognition of a considerable phenotypic variation among populations. However, despite the large variation in body size, sexual dimorphism, body scalation, and coloration observed, a low level of genetic diversity was detected among populations [18]. Likely the earliest genetic divergence among *P. pityusensis* populations took place around 0.18–2.29 Ma [18]. The fragmentation of the western Pityusic islands, collectively known as the Bledas Islands, occurred around 14,000 years ago.

In this scenario, several translocations between the populations of *P. pityusensis* took place, even in recent times [19]. Thus, the migration of gene copies from the Formentera Island to the Ibiza Island and some coastal islets has been detected, showing the existence of mitochondrial captures after the introduction of individuals from the Formentera Island [19]. However, the offshore islets from the western Ibiza coast appear to maintain clearly differentiated genetic traits with respect to these translocations from Formentera. This fact may have significant importance for the conservation genetics of the species, since the populations of the most remote western islets of Ibiza would be isolated from mitochondrial colonisation originating from the populations of the two main islands (Ibiza and Formentera) and, for this reason, it would be the best representation of the genetic composition of the original *P. pityusensis* lineage [19].

**Figure 1:**
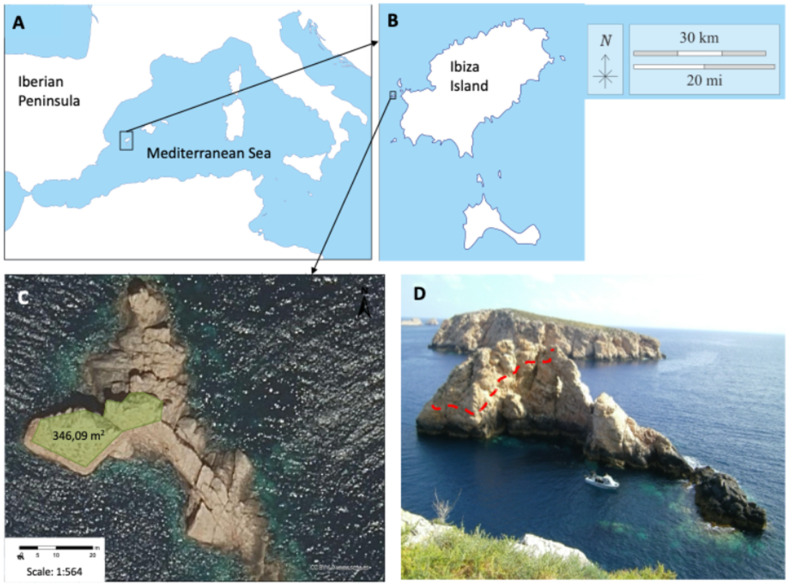
(**A**) Location of the Pityusic Islands in the western Mediterranean. (**B**) Location of Vaixell Islet on the western coast of Ibiza (from https://d-maps.com/carte.php?num_car=3146&lang=es and https://d-maps.com/carte.php?num_car=13428&lang=es, accesed on 30 July 2025). (**C**) Satellite image of Vaixell Islet. In pale green, boundaries of the approximate area with scarce vegetation cover where lizards are present (346.09 m^2^). Image and surface calculations were obtained from SignA (Sistema de Información Geográfica Nacional, Instituto Geográfico Nacional, accessed on 28th July 2025). (**D**) Southern slopes of Es Vaixell Islet seen from Na Gorra Islet. The red dotted line approximately delimits the area covered by sparse shrub vegetation (see also (**C**)). The rest of the islet is rocky, without vegetation, lacking suitable shelters for lizards, and battered by the sea during storms.

**Figure 2:**
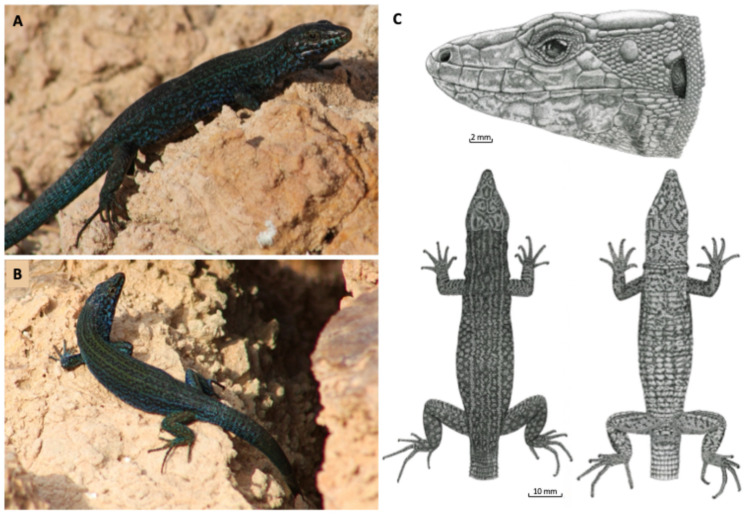
(**A**) Adult male of *P. pityusensis* from es Vaixell. (**B**) Adult female. See in both cases the blueish nuances typical of lizards from this population. (**C**) Details of head scalation and patterns of dorsal and ventral views of an adult lizard (drawings from Ana Pérez-Cembranos).

In 2010, during the annual survey regarding the conservation status of the Pityusic wall lizard, a new population was detected at the western Ibiza coast at Vaixell Islet [20,21]. Vaixell probably became isolated from the nearest island, Na Gorra (Figure 1D), around 8500 years ago [22]. In two previous works [20,21], we analysed the status of this population, its relationship with the remaining populations of the Pityusic wall lizard, and the story of the population in relation with lizard introductions made by the German herpetologist, Martin Eisentraut, during the first third of the 20th century. Eisentraut [23] introduced 51 lizards from the Ibiza Island to Vaixell but, apparently, none reproduced or survived [20,21]. Paradoxically, the Vaixell lizard population is an excellent example of a population “not contaminated” by translocations, despite having suffered this episode of deliberate introduction which, fortunately, was not successful. The design and coloration of lizards from Vaixell were already described, pointing out that it is a melanistic population of very large lizards (Figure 2 and [21]). Among the populations of *P. pityusensis*, lizards from Vaixell showed the largest body sizes [15,21].

In this study, we examine how the characteristics of Es Vaixell Islet influence the natural history and ecological traits of its lizard population. We want to explore the adaptive capabilities of an original population of *P. pityusensis* that has not been contaminated by other populations and that has remained in extreme isolation for thousands of years, subjected to the selective pressures resulting from the extremely limited habitat and the radical scarcity of trophic resources. We describe the basic demographic characteristics of this unique population, with an estimation of its population size and the patterns of body growth. After nine visits occurring in different years, we are ready to present some aspects of the natural history of this population in the medium term. We analyse the population density, as well as its adult sex ratio (ASR) and their annual variations, the intensity of missing toes and autotomized tails. We also study the diet and how these lizards grow and reach such large body length. Our main hypothesis is that the Pityusic wall lizard has adapted to extreme conditions of this very small islet due to it being the only terrestrial vertebrate. Our prediction is that the abundance, adult body size, sex ratio, and diet are the result of adaptive processes shaped by the islet’s conditions. In particular, the growth rate of individuals could be a response to the lizards’ ecological conditions, especially during their juvenile phase.

## 2. Materials and Methods

### 2.1. Study Site and Sampling

Es Vaixell (38° 58″ 54.5″ N, 1° 10′55.9″ E, western Ibiza, the Balearic Islands, Spain) is an islet of a very reduced surface. Lizards occupy an even more reduced area characterized by almost vertical slopes and very difficult access without climbing equipment (Figure 1C,D). Despite this, during visits to the islet, we tried to reach and sample the same areas on all occasions, including all surfaces with some shrub vegetation. It is difficult to establish the surface covered by plants, but we can roughly estimate that plants cover around 346 m^2^ (Figure 1C). Within this area, we restricted our lizard sampling to a 146 m^2^ zone, the only surface accessible for capturing and recapturing lizards. The remaining area, covered by some vegetation, is extremely steep, making safe capture and recapture efforts extremely difficult. Our objective was to standardise the sampling effort on each occasion. In this way, we have tried to ensure comparable visits in terms of capture and recapture probabilities, although the conditions of the islet were far from optimal for such an objective. Lizards were captured using a noose. Upon capture, we sexed each lizard and measured its SVL, body mass, and tail length, and we determined if the tail was regenerated after an autotomy event. In addition, we recorded the number of missing toes. After measurements and identification (see below), lizards were released at the site of capture (see more details in [21]).

**Figure 3:**
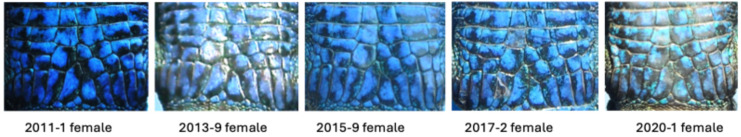
An example of digital images of an adult female captured in five different years on Vaixell Islet. Despite different lighting conditions, Wild-ID software recognised the same individual on each occasion.

The orography of the islet (Figure 1D) and working conditions have prevented line transects from being carried out. We have therefore chosen to carry out an estimate of the density of lizards using a capture–recapture programme based on nine visits to the islet: in June 2011, 2013, 2014, 2015, 2016, 2017, 2019, and 2021, as well as one visit in September 2020. Lizards were photographed using a DSLR system, a Nikon D90 camera (Nikon, Ayutthaya, Thailand) with a 60 mm macro lens. During each visit, lizards were identified through digital photographs of the ventral area, which were then compared using the Wild-ID free software (ver. 0.9.31) [24]. Wild-ID uses the SIFT operator to detect the features of each image and compare them across a large dataset [25]. In our case, the arrangement of the ventral scales of the first five to six rows, immediately behind the collar scales, had a unique pattern for each individual lizard, allowing their identification during each capture–recapture session (Figure 3).

The capture and recapture data have been analysed using the “Rcapture” software (ver. 1.4-4) [26] in the R environment (ver. 4.5.1) [27], which allowed the analysis of the capture histories of each specimen through log-linear models. The data were arranged as capture histories for captured lizards. Each capture event in the experiment was recorded using format 1 of Rcapture [28], where each row represents an observed capture history. The rows contain only zeros (no captured) and ones (captured). The number of columns in the table represents the number of capture occasions and the number of rows the number of captured lizards during the experiment (Table A1).

Rcapture uses Poisson regressions to estimate parameters in the capture–recapture experiment [28]. Rcapture can fit a total of three general model types to the data. Based on our capture and recapture data, we considered the study of an open population. We employed the function “openp”, setting it to “up” (capture probabilities vary between periods, up = unconstrained probabilities), because capture occasions are widely spaced in time, around 1 year or more apart. We considered the captured lizards to be a random sample of the lizards in the population at a given capture occasion [28]. Thus, we applied a Jolly–Seber model, because identified and unidentified lizards undergo the same sampling process and population sizes and survival rates can be estimated. The Rcapture package follows the log-linear approach of Cormack [29] to fit the Jolly–Seber model [28]. We employed the common growth rate test [28] to verify if the population is growing or decreasing at a constant rate over time. We detected only three subadult lizards (SVL < 70 mm) during the visit in September 2020. During the remaining visits, we captured only adult lizards. We calculated the adult sex ratio (ASR) of lizards on each visit to Vaixell. Sex assignment has been carried out thanks to sexually dimorphic characteristics such as body size, head size, and the development of femoral pores in males and females. The proportion of adult males and females captured on each visit to Vaixell was tested using the G test of the RVAideMemoire package (ver. 0.9-83-12) [30].

### 2.2. Body Growth

During the sampling years, we only obtained recapture data for a total of 26 individuals. Eleven lizards were recaptured on more than one occasion. On each occasion, the snout–vent length (SVL) of the lizard was measured to the nearest 0.5 mm by laying the lizard flat along a steel rule. SVL is more commonly used in lizards’ studies of body growth because it is less sensitive to temporary variations in body condition than body weight [31]. We approached the study of body growth with this small sample that only included three individuals who, judging by their size (see above), could be considered subadults at the time of their first capture. A csv file has been built with all the recaptured individuals, including their identification (id), sex, SVL at the time of the first capture (l_1_), SVL at the time of the second or subsequent recaptures (l_2_), and the time elapsed between recaptures (dt), calculated in years, with decimals corresponding to the additional months.

We tried to fit three different growth models (Table 1)—a von Bertalanffy model with the Fabens modification [32] since we did not know the age of the lizards at the time of their first capture; A logistic model that was previously employed in several studies on lizard body growth [31], and the Gompertz model [33]. The Fabens modification uses the increments in body size of an individual in successive captures to estimate its growth rate. In our case, we included a SVL of 32 mm at birth. This body size corresponds to one of the upper limits of body sizes at birth observed in *P. pityusensis* [34], (see also [35,36]), since the population from Vaixell has the largest adult body sizes observed in this species [21]. In addition, we observed an adult male with an SVL of 93.5 mm, which was subsequently recaptured. So, we included an asymptotic maximum length of L_∞_ = 95 mm in the growth models.

To fit the three growth models, we employed the R package “nlme” (ver. 3.1-168) [39], which allows the analysis of captures and recaptures in a format in which each recapture occasion is represented as a row. The three models were fitted using the maximum likelihood (ML) of the nlme package. One of the advantages of the nlme package is that it allows the analysis of unbalanced individuals, that is, individuals with an unequal number of recaptures, which also occurred at irregular intervals. In the three cases, we considered nonlinear mixed models [40] with fixed effects (sex) and random effects (individual lizards). The models were then compared using the Akaike information criterion (AIC) and the LogLik of each fitted model. This resulted in the selection of the most parsimonious model, excluding all factors without a statistically significant influence.

### 2.3. Diet Analysis

Dietary information regarding Vaixell lizards was obtained from 61 faecal samples collected from 2011 to 2021. We have considered the June samples as spring samples and the July and September samples as summer samples. Faeces were directly obtained from the ground or from captured lizards that defecated during handling. We analysed the faecal samples under a binocular dissecting microscope. Diet reconstruction based on meticulous faecal pellet analysis has been found to be highly comparable to diet reconstructions based on gastric contents removed from dissected stomachs, with soft-bodied prey and particularly insect larvae being equally represented in faecal pellets and gut contents [41]. Furthermore, faecal pellet analysis is a standard method for quantifying diet with the added advantage of not compromising animal well-being. Each individual faecal sample was spread in a thin layer of less than 0.5 mm over the entire surface of a Petri dish with several drops of 70% ethylic alcohol. The percentage of vegetal matter was then visually estimated according to the surface occupied by vegetal remains in the Petri dish. Prey remains were identified up to their order level or, exceptionally, to the family level. The prey number for each faecal pellet was conservatively estimated by counting only identifiable remains. The consumption of bird and mammal carcasses was inferred from the presence of bones, feathers, or hairs, and hair identification was performed employing the work of Teerink [42].

The diet was described in terms of the relative contribution of each prey item. We calculated prey abundance (%n) as the percentage of a given prey type in relation to the total prey number, and we calculated relative prey or plant presence (%p) as the percentage of faeces containing given prey type or plant. The spring and summer diets of lizards were compared via permutational multivariate analysis of variance (permutational MANOVA [43]) with the ‘adonis’ function from the ‘vegan’ R package (ver. 2.7-1) [44]. The multivariate homogeneity of group dispersion (variances) was tested the ‘betadisper’ function from the ‘vegan’ package, a multivariate analogue of Levene’s test for homogeneity of variances. We estimated and compared diet diversities employing the approach proposed by Pallmann et al. [45]. Instead of describing diet diversity through a given index, we converted these “raw” indices into “true” diversities, which all belong to one and the same mathematical family, that is, different measures were considered to be special cases of Hill’s general definition of diversity measures [46]. In this way, to study differences in diversity between spring and summer diets, we performed two-tailed tests based on integral Hill numbers. This selection included the transformed versions of the three following indices: the species richness index, H_sr_ (q = 0); the Shannon entropy index, H_sh_ (q → 1); and the Simpson concentration index, H_is_ (q = 2, [47]). We performed 5000 bootstrap replications to obtain reliable *p*-values. The methods described here are implemented in the R package “simboot” (ver. 0.2-8) [48]. Owing to the non-normality of distribution in samples (Shapiro–Wilk test, W = 0.55455, *p* = 2.521 × 10^−12^), the percentages of plant matter in the diet were compared using a Kruskal–Walli’s test. All calculations were performed in R [27].

## 3. Results

### 3.1. Plant Cover

The vegetation of Vaixell is very reduced but not absent. As main plant species, we detected *Limonium ebusitanum*, *Lavatera arborea*, *Arthrocnemum macrostachyum*, *Asparagus horridus*, *Halimione portulacoides*, and an unidentified Graminaceae. Small shrubs are isolated in protected crevices and close to large rocks, representing excellent refuges for lizards (Figure 1D).

### 3.2. Abundance

We sampled the population only once each year. Thus, we were unable to construct the model of the population in a hierarchical way [28]. We obtained data from 68 lizards. Fitting a Jolly–Seber model of open populations, we obtained a deviance of 100.656 with 489 degrees of freedom and the AIC of 216.451. The test for trap effect showed a homogenous trap effect during the study (model with homogenous trap effect: deviance = 99.316 with d.f. = 488 and AIC = 217.111; model with trap effect: deviance = 90.832 with d.f. = 483 and AIC = 218.628). In the studied area of Vaixell (146 m^2^), depending on the year, we estimated that between 21.6 ± 8.4 and 48 ± 23.2 lizards inhabited the site; therefore, we can estimate that between 50 and 114 lizards could live on Vaixell Islet (Table 2). We only have observations of subadult individuals from September 2020, so we lack information on the proportion of subadults in this population. Our abundance estimates correspond to annual densities of 1438 to 3288 individuals per hectare. Testing the hypothesis of a common growth rate of the population, we obtained *χ*^2^ = 1.7507, *p* = 0.4167. Thus, we do not reject the hypothesis of common annual growth rate im the population of lizards over time. This indicates that the model with a constant growth rate is statistically adequate for describing the dynamics of the studied population.

### 3.3. Injuries, Sex-Ratio, and Body Growth

A very high proportion of males (70.59%) and females (77.77%) showed a regenerated tail, without significant differences between sexes (Fisher test, *p* = 0.5882). In addition, more than a half of males (56.67%) and females (51.51%) had at least one injured toe, again without significant differences between sexes in terms of injury frequency (Fisher test, *p* = 0.8014). Adult sex ratio (ASR) was equilibrated during all years of research (Table 3).

The fitting of the Gompertz model was better than the fitting of the logistic and von Bertalanffy models (Table 4), thus, the Gompertz model was selected. The comparison of models with or without sex as a fixed effect indicated that the model including the sex of individuals provided a better fit to our data (model without the sex: AIC = 241.1252, LogLik = −116.56259; model with the sex: AIC = 211.2829, LogLik = −99.64146; L. ratio test = 33.84226, *p* < 0.0001). Thus, it can be concluded the sex of the lizard has a significant influence on its body growth. The QQ normality plot and the Shapiro–Wilk normality test (W = 0.91392, *p* = 0.001617) indicate that residuals are not normally distributed. To address this problem, we incorporated the variance-power function (varPower) from the nlme package into the model, using initial size (l_1_) as a covariate. In this way, the robust Gompertz model fully met the assumptions of the normality of the residuals (Shapiro–Wilk normality test, W = 0.96433, *p* = 0.1431) and showed a better fit, according to the Akaike information criterion (AIC = 201.5602). In this robust model, the growth coefficient of variation, σ^2^ = − 4.162242, is an estimation of the degree of growth heterogeneity. This negative value indicates a reduction in residual variance with the increase of SVL. In other words, juveniles are much more variable than adults in their growth rate.

**Figure 4:**
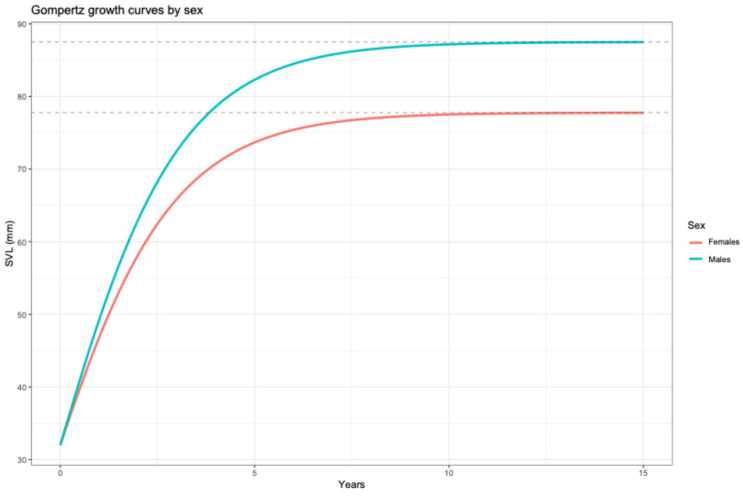
Gompertz growth curves of females and males of *P. pityusensis* from Vaixell Islet. Dotted horizontal blue and red lines indicate the maximum asymptotic values of curves.

Finally, although the separation of the growth curves (Figure 4) was significantly different in males and females (Table 5, *p* < 0.05), indicating a clear sexual dimorphism in their final body size and different asymptotic sizes, there is no evidence that the relative growth rate differs between the two sexes (t = −0.49828, *p* = 0.6237).

Therefore, we can simplify the final model by considering a common g value for males and females. In the Gompertz model, g indicates the deceleration of growth; that is, it defines the curvature of its growth curve and indicates how quickly the lizard goes from its maximum growth to the slow growth phase. According to the final Gompertz model (Table 5), males and females of *P. pityusensis* from Vaixell have similar characteristic growth rates (g). The final model (Table 5) is preferable to the previous robust model (robust model (AIC = 201.5603, LogLik = −93.78016; final model, AIC = 199.5412, LogLik = −93.77059), and this simplification is statistically valid (L. ratio test, *p* = 0.8899).

**Figure A1:**
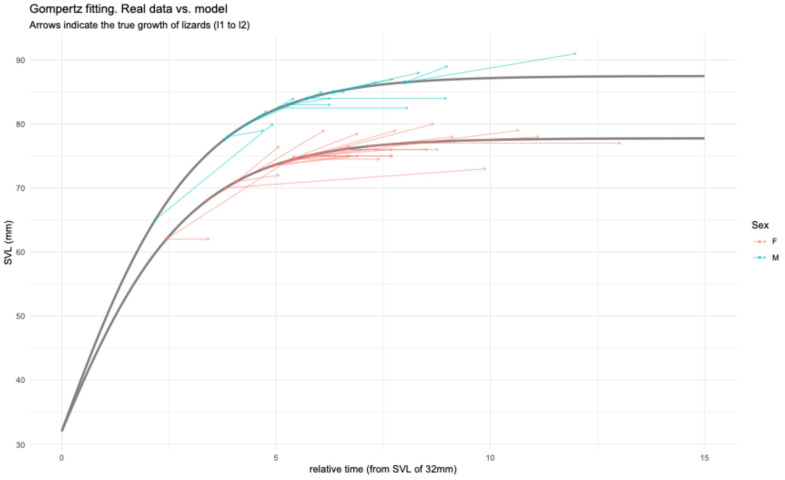
Gompertz growth curves for males and females with the trajectories of real growth of individuals.

In this population of *P. pityusensis*, we do not know the size of the lizards at sexual maturity (L_m_), but we can estimate the SVL of the lizards at the inflexion point of the growth curve (L_i_) [47]. In the Gompertz model, L_i_ corresponds to the moment of maximum growth efficiency, that is, the moment when growth transitions from acceleration to deceleration. In the Gompertz model, this moment theoretically occurs when the individual reaches 36.79% of its asymptotic size [33], therefore L_i_ = L_∞_/e. If we consider a hatching size of 32 mm, our results indicate that the peak growth acceleration occurs prenatally or that it practically coincides with the moment of hatching. The maximum theoretical SVL at the inflexion point of the curve (L_i_) is L_i (females)_ = 28.6024 mm and L_i (males)_ = 32.1880 mm (Table 5). Biologically, this would imply that males and females are born operating in the deceleration phase of their ontogenetic growth curve. The Gompertz model allows to calculate the absolute growth rate as C_max_ = (g·L_∞_)/e [33], that is, the highest estimated annual growth. Thus, males hatch having reached their maximum potential growth rate (C_max males_ = 18.006 mm/year; Table 5), while females are born having slightly exceeded this metabolic threshold (C_max females_ = 16.003 mm/year; Table 5). Although adult males from Vaixell exhibit a higher maximum growth rate than females, the difference between the two sexes is not statistically significant (Table 5; Figure A1).

### 3.4. Diet

**Figure 5:**
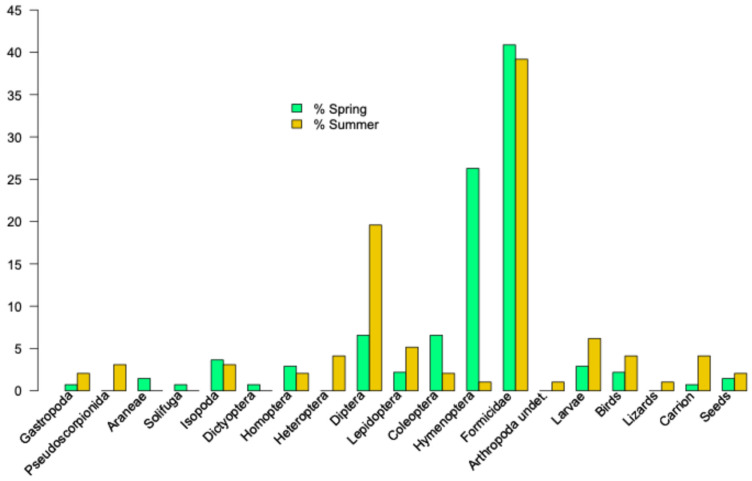
Spring and summer diets of *P. pityusensis* from Vaixell Islet (see more details in the text).

The diet is dominated by the consumption of clumped prey, particularly ants (spring and summer diets, Figure 5). Some differences are interesting. Hymenoptera were present mainly during spring, whereas Diptera showed the opposite trend (Table A4 and Figure 5). This result revealed that diets did not differ between spring and summer (permutational MANOVA, F = 0.9977, *p* = 0.386).

In comparing Hill’s numbers across seasons, we did not detect significant differences between the summer and spring dietary diversities for the three Hill’s numbers—q = 0, *p* = 0.6382; q = 1, *p* = 0.7170; and q = 2, *p* = 0.07536. The low average plant matter volume was also similar in spring and summer (spring: $\bar{x}$ = 16.06 ± 5.49%, *n* = 33; summer: $\bar{x}$ = 17.86 ± 6.72%, *n* = 28; Kruskal–Wallis test, *χ*^2^ = 0.07478, *p* = 0.7845).

## 4. Discussion

On Vaixell Islet, we have a population of large lizards living on a very small islet, with a significant lack of available resources and an absence of terrestrial predators. In this situation, indicators of potential predation pressure acquire relevance. In this sense, we interpret the high incidence of missing toes observed in lizards from Vaixell as a direct reflection of aggressive interactions between individuals. Vervust et al. [48] consider the incidence of missing toes to be exceptionally high (55.48% of individuals) in the *Podarcis siculus* population of Pod Mrčaru (Croatia). We have already seen that our incidence values in males from Vaixell are even higher. This seems to be the most plausible interpretation in the case of the Pityusic wall lizard, as also observed in the Italian wall lizards studied by Vervust et al. [48]. Consequently, high population density could promote intraspecific competition. Perhaps the most surprising finding is the absence of differences in the proportion of missing toes between males and females, which indicates that adult females are involved in aggressive encounters just like males. On Vaixell Islet, strong predator pressure does not appear to exist (see above), and yet we find a very high rate of autotomized tails, similar in males and females, which, given the high population density, can also be interpreted as a reflection of frequent aggressive interactions. Even if the high proportion of autotomized tails has traditionally been interpreted as an index of predation pressure [49,50], this interpretation is problematic, as Schoener pointed out [51]. In *Podarcis gaigeae* from the Skiros Archipelago (Greece), where predation pressure is relaxed, the proportion of regenerated tails has also been considered as reflecting high levels of intraspecific aggression [52].

On Vaixell Islet, as in most of the populations of *P. pityusensis* (unpub. results), there is an equilibrated adult sex ratio (ASR), with only an annual random variability. Es Vaixell has a high lizard density, which usually happens in these small islets of the Balearic Archipelago within the two endemic species of lacertid lizards [6,10]. However, this density translates into a reduced population size due to the small surface available to lizards. In addition, these lizards have largest body sizes among all known populations of the Pityusic wall lizard [21]. Such body sizes correspond to individuals of notable longevity, with specimens that are more than 10 years old. This fact indicates that, in such extreme conditions, it is very probable that a strict selection process takes place, resulting in the survival of large individuals that reach significant longevities. In captivity, longevity of around 18 to even 30 years was observed in males and females of *P. pityusensis* [53,54]. Moreover, the tendency towards larger body sizes is characteristic of the small coastal islets of Ibiza, among which Vaixell is no exception [21]. Case [2] discussed the importance of environmental factors on the body size of insular populations, relying, in the case of lacertid lizards, on the studies of Mertens [55], Kramer, and Mertens [56,57]. In insular reptiles, food availability appears not to be a sufficient factor for explaining the observed body sizes, so the absence of predators has to be considered [2]. Meiri [12] specifically points out that the absence of mammalian predators could be the fundamental factor for larger body sizes in species of the genus *Podarcis*.

In reptiles, there are highly plastic growth patterns dependent on environmental factors such as food availability and temperature, reflecting an adaptive strategy [58]. In theory, the Gompertz model could better describe the growth of turtles and crocodiles and may be less suitable for lizards and snakes, whereas the von Bertalanffy model would be a better option [59]. For the lizards of Vaixell, the best fit is obtained with the Gompertz model. We have observed that sex significantly affected asymptotic size, but no differences were found in growth rate (g) between males and females. A similar result was observed in *Anolis* lizards from Bahamanian populations [31]. Working on Moltona Islet (Mallorca, Balearic Islands) with *Podarcis lilfordi*, the sister species of *P. pityusensis*, Rotger et al. [60] found a similar k growth parameter in males and females of *P. lilfordi*. These authors showed that the final asymptotic body size was, as in our case, different in males and females.

The final Gompertz model accumulated most of the AIC weight (w_i_ > 0.90, Table 5), indicating a clear superiority over the other two fitted models that do not adequately describe the growth of this species. The asymptotic size estimated by the Gompertz model for males (SVL = 87.49 mm, Table 5) is slightly lower than the maximum size observed in the population (SVL = 93.5 mm of one individual male). This result is expected in a mixed-effects model in which L_∞_ represents the population average and not the maximum physiological limit of the species. We can conclude that adult males reach significantly larger body sizes than females (Table 5).

It was established that the growth of insular lizards has a very rapid juvenile phase after hatching, and then it declines monotonically. Although post-hatching growth is generally studied, it is a continuation of an embryonic growth curve [58]. In the lizards of Vaixell, when fitting the Gompertz model, the inflexion point is mathematically located very close to, before, or after hatching. In our case, most of the metabolic acceleration occurred before hatching, with the maternal yolk being consumed in the egg. In *P. pityusensis* and other lizard species, the residual yolk in the egg contributes very little or nothing to the post-embryonic growth of juveniles [61].

In their general body growth model, West et al. [62] point out that the slowing of growth is a consequence of the way organisms distribute energy. Although their study only includes endotherms (birds and mammals) and fish-like ectotherms, they demonstrate that growth begins to slow within the egg when the cost of forming and maintaining tissues equals the circulatory system’s capacity to supply nutrients. In lizards, eggs are highly dependent on humidity, so, as the embryo grows, the egg swells by absorbing water, the shell tightens, and gas conductance changes [63,64]. The embryo then reaches its critical size, at which its oxygen demand exceeds its supply capacity. At this point, growth slows. For example, Thompson and Stewart [64] demonstrated in *Eumeces fasciatus* that the oxygen consumption of the embryo increases very rapidly until day 15 of incubation, but around day 21 it slows down and, although growth continues, it is significantly slower. This strategy suggests a maximum energy allocation during embryonic development and the very early postnatal life stages, which likely minimises the time spent at the sizes most vulnerable to predation. However, the lizard population of Vaixell does not appear to experience significantly higher predation pressure than other nearby populations of *P. pityusensis*. But, in this population, we can consider conspecifics as terrestrial predators, even if such predation cases were occasional.

The diet of the lizards from Vaixell is like the diet described in several populations of *P. pityusensis* [65]. Although the area with vegetation covers about 346 m^2^, the lizards move even through low areas of the islet, reaching the water’s edge where they capture marine isopods such as *Ligia italica*, as is the case in other populations of *P. pityusensis* (unpublished data). In a previous metabarcoding analysis that included some Vaixell faeces [66], we identified *Ligia italica*, as well as a terrestrial isopod, *Halophiloscia hirsuta*, a common species in Balearic Islands that lives under stones, even in areas without vegetation [67,68]. The opportunistic nature of the lizards of Vaixell is evident not only in their consumption of these isopods, but also in their capture of flies such as *Acartomyia mariae*, a Culicidae whose larvae develop in brackish pools [69], like those that appear on coastal islets after storms. The metabarcoding analysis also detected the presence of an unidentified orthopteran from the family Tettigonidae [70]. Lizards in this population consume less than half the volume of plant material compared to other populations from the coastal islets of western Ibiza [18] (and unpublished data). This is probably the result of a very limited availability of plant matter, especially fleshy fruits from shrubs. Only fruits from *Asparagus horridus* [18] were detected. In fact, the DNA from plants was absent in our previous metabarcoding study [66].

Despite the small sample of faeces analysed, we recorded the presence of a juvenile individual in the summer diet of *P. pityusensis* from Vaixell (Figure 5 and Table A4). In fact, “partial cannibalism”, that is, the consumption of tails by conspecifics, has been reported in Pityusic wall lizards kept in captivity [71]. Cases of cannibalism are frequent in populations of *P. pityusensis* [72,73] and several insular populations of lacertid lizards [74] (and references therein)]. Thus, it is likely that lizards from Vaixell do not suffer greater predation pressure from avian predators than other populations (unpublished data), but the scarcity of resources and, in general, the extreme conditions under which this small population lives may result in a greater predation pressure from conspecifics, increasing the cases of cannibalism against smaller individuals. This selective pressure may have shaped growth rates with peaks before hatching as an adaptive strategy for quickly reaching body sizes that would allow newborns to avoid this conspecific predation pressure. Of course, due to the frequent presence of cannibalism in many other populations of the Pityusic wall lizard, we cannot rule out that this strategy is common to other populations. Studying five populations of *Podarcis gaigeae* with differences in predation pressure, lizard density, and seabird presence, Pafilis et al. [7] noted that larger adult body sizes were observed in populations with higher lizard density and lower predation pressure. Larger adult lizards are found in populations where juveniles are born with a larger body size, increasing their chances of survival against predation by conspecifics [2].

The Pityusic wall lizard is characterised by an extraordinary phenotypic variability, encompassing not only the most obvious morphological features, such as coloration, pattern, and body size, but also less conspicuous traits like behavioural, ecological, and natural history characteristics [4]. This variability likely originated from the ancestral mixing of genomes that underwent introgression from lineages that had evolved independently for millions of years [75]. It is therefore not surprising that, in this scenario, isolated populations with particular morphological and ecological features appear, especially if environmental conditions have acted as drivers of adaptive changes. A genetic analysis showed that the lizards from Vaixell are remarkably close to lizards from Na Gorra, a nearby islet with which they shared a common ancestor [21]. Lizards from Vaixell were later arbitrarily assigned to the subspecies *Podarcis pityusensis gorrae*, without any systematic or morphological study and, in fact, without any observation or even a single specimen [76]. However, here, we do not try to discuss the subspecific status of lizards of Vaixell, because in the case of the Pityusic wall lizard, our point of view is that the description and enumeration of subspecies should be abandoned and replaced with the recognition of evolutionary significant units (ESUs), as we proposed in the case of the Lilford’s wall lizard, *Podarcis lilfordi* [77].

Vaixell is a very illustrative case of life on very small islets of the Mediterranean and, for this reason alone, this population deserves strict protection. The most advisable course of action would be to completely avoid landing on this population, only allowing annual monitoring and avoiding disturbing the delicate balance of this small population of lizards living in such extreme conditions. Es Vaixell is an extraordinary example of adaptation, not only to the harsh environmental conditions of the islet, but also an example of resistance to human pressure, represented here by Eisentraut’s regrettable experiment conducted almost 100 years ago. The survival of the native lizards and the disappearance of the introduced ones indicate that the ecological and natural history traits of the native population are adaptive and that they are not simply the product of genetic drift in a population with a very small number of individuals.

## 5. Conclusions

Aside from the recent confusing and even contradictory information about the lizard population of Vaixell, this islet is home to a native population of the Pityusic wall lizard. Lizards show an exceptional body size and survive in extreme environmental conditions thanks to their adaptation over thousands of years. In this environment, there is intense intraspecific competition involving both males and females, with a predatory pressure from adult individuals on juveniles that has translated into strong body growth rates and great body sizes at birth, as an adaptation technique for escaping intraspecific predation. As in its sister species, the Lilford’s wall lizard, the Pityusic wall lizard populations are excellent examples of the adaptive capacities of island lacertids in the face of the challenges of life on tiny islets.

## Figures and Tables

**Table 1:** Fitted body growth models and references for their application to capture and recapture data, where l_2_ is the expected body size after the time interval between recaptures; L_∞_ is the asymptotic maximum body size; l_1_ is the size observed at the first capture; g is the growth rate decay coefficient in Gompertz model; k is the growth rate in logistic and von Bertalanffy models; and dt is the time elapsed between two recaptures (see more details in the text).

Model	Equation	References
Gompertz	l_2_ = L_∞_ · (l_1_/L_∞_)^(e(−g·dt))^	[33,37]
Logistic	l_2_ = (L_∞_ · l_1_)/(l_1_ + (L_∞_ − l_1_) · e^(−k·dt)^)	[31]
von Bertalanffy	l_2_ = L_∞_ − (L_∞_ − l_1_) · e^(−k·dt)^	[32,38]

**Table 2:** The results of the analysis of capture–recapture data of lizards from Es Vaixell Islet obtained employing a Jolly–Seber model of open populations (capture probabilities and abundances for each period ± SE) and survival probabilities between periods (±SE) (see more details in the text).

Periods	Capture Probabilities (Estimate ± SE)	Abundances (Estimate ± SE)	Between Periods	Survival Probabilities (Estimate ± SE)
1	--	--	1–2	1 ± 0.00
2	0.2370 ± 0.0969	67.5 ± 23.3	2–3	0.7417 ± 0.2598
3	0.2697 ± 0.1447	49.9 ± 23.4	3–4	0.5531 ± 0.1759
4	0.5310 ± 0.1623	32.0 ± 8.2	4–5	0.8897 ± 0.2171
5	0.4848 ± 0.1511	35.1 ± 9.1	5–6	0.8913 ± 0.4001
6	0.1739 ± 0.0924	40.2 ± 16.3	6–7	0.5925 ± 0.2844
7	0.3774 ± 0.1390	23.8 ± 6.1	7–8	0.9049 ± 0.3950
8	0.3244 ± 0.1616	21.6 ± 8.4	8–9	0.7857 ± 0.4698
9	0.4091 ± 0.2323	31.8 ± 16.7	9–10	--

**Table 3:** Adult sex ratio of *P. pityusensis* from Vaixell Islet. We include the G tests and their corresponding *p*-values.

Year	ASR	G	*p*-Value
2011	0.55	0.20033	0.9775
2013	0.5385	0.0769	0.9944
2014	0.4118	0.53219	0.9118
2015	0.3684	3.3938	0.4942
2016	0.2857	1.3283	0.7224
2017	0.5454	0.18207	0.9804
2019	0.3861	0.69859	0.8735
2020	0.4444	0.33402	0.9535
2021	0.4090	0.73133	0.8658

**Table 4:** Model fitting comparison. Fixed parameters are identical for the three models, as well as K(n): the number of estimated parameters, including fixed effects, the variance of the random effect, and the residuals. For each model we provide the Akaike information criterion (AIC) and the ΔAIC, that is, the difference between the AIC of each pair of models compared. Because ΔAIC is >10 for logistic and von Bertalanffy models, in comparison with the Gompertz model, we can conclude that the Gompertz model is significantly superior; w_i_ (AIC weights) is the probability that a model is the best in the comparison. LogLik is the value of the likelihood function that indicates the degree of model fitting before the inclusion of parameters.

Model	Fixed Parameters	K(n)	AIC	ΔAIC	w_i_ (AIC Weight)	LogLik
Gompertz	L_∞ (sex)_, K_(sex)_	5	199.54	0.00	0.9944	−93.77059
Logistic	L_∞ (sex)_, K_(sex)_	5	210.94	11.40	0.0033	−99.4791
von Bertalanffy	L_∞ (sex)_, K_(sex)_	5	211.67	12.13	0.0023	−99.83608

**Table 5:** Fitting of the Gompertz model for males and females with a common growth rate (g). L_∞_ is the asymptotic maximum body size. For each parameter, we give its estimation ± SE, the 95% confidence intervals (CI 95%), and *t* tests with corresponding *p*-values. In the case of C_max_ values of males and females, we did a Wald test, showing that, even if males showed a higher C_max_, this difference is not statistically different (see also the wide overlap of 95% confidence intervals).

Parameter	Sex	Estimate	±SE	CI 95%	*t* and *p*-Values
L_∞ (Intercept)_	♀♀	77.76529	0.7797302	[76.1942, 79.3364]	99.73, *p* < 0.0001
L_∞ (males)_	♂♂	9.7307	1.1563173	[7.4008, 12.0606]	8.41, *p* < 0.0001
g	common	0.55939	0.0969641	[0.364, 0.7548]	5.76, *p* < 0.0001
C_max_	♀♀	16.003	2.651	[10.519, 21.487]	0.505, *p* = 0.61
	♂♂	18.006	2.951	[11.901, 24.110]	

## Data Availability

The data presented in this study are available in Table A2 and Table A3 of the Appendix A.

## Abbreviations

The following abbreviations are used in this manuscript:

ASR	Adult Sex Ratio
AIC	Akaike Information Criteron
ESU	Evolutionary Significant Unit
MANOVA	Multivariate Analysis of Variance
ML	Maximum Likelihood
SVL	Snout–Vent Length

## Appendix A

**Table A1:** Frequency statistics from capture–recapture histories: f_i_ = number of lizards captured i times; u_i_ = number of lizards captured for the first time on occasion i; v_i_ = number of lizards captured for the last time on occasion i; n_i_ = number of lizards captured on occasion i.

Occasions	f_i_	u_i_	v_i_	n_i_
i = 1	43	20	11	20
i = 2	14	9	7	13
i = 3	3	10	6	17
i = 4	5	7	9	17
i = 5	2	3	4	7
i = 6	1	3	4	9
i = 7	0	1	4	7
i = 8	0	7	10	13
i = 9	0	8	13	13

**Table A2:** Capture–recapture histories of lizards from Vaixell Islet during the study period.

2011	2013	2014	2015	2016	2017	2019	2020	2021
1	0	0	1	0	0	0	0	0
1	1	1	1	0	0	0	0	0
1	0	0	1	0	0	0	0	0
1	0	0	0	0	0	0	0	0
1	0	0	0	0	0	0	0	0
1	1	0	1	0	1	0	1	0
1	0	0	0	0	0	0	0	0
1	1	1	1	0	0	1	0	0
1	0	0	0	0	0	0	0	0
1	1	1	1	0	0	0	0	0
1	0	0	0	0	0	0	0	0
1	0	0	0	0	0	0	0	0
1	0	1	0	0	0	0	0	0
1	0	0	0	0	0	0	0	0
1	0	0	0	0	0	0	0	0
1	0	0	0	0	0	0	0	0
1	0	0	1	0	0	0	0	0
1	0	0	0	0	0	0	0	0
1	0	1	0	1	0	0	0	0
1	0	0	0	0	0	0	0	0
0	1	1	0	0	0	0	0	0
0	1	0	0	0	0	0	0	0
0	1	0	0	0	0	0	0	0
0	1	0	0	0	0	0	0	0
0	1	0	0	0	0	0	0	0
0	1	0	0	0	0	0	0	0
0	1	0	0	0	0	0	0	0
0	1	1	0	0	1	1	1	1
0	1	0	0	0	0	0	0	0
0	0	1	1	0	0	0	0	0
0	0	1	1	0	1	0	0	0
0	0	1	0	0	0	0	0	0
0	0	1	1	0	0	0	0	0
0	0	1	0	0	0	1	0	0
0	0	1	0	1	0	0	1	0
0	0	1	0	0	0	0	0	0
0	0	1	0	0	0	0	0	0
0	0	1	0	1	0	0	0	0
0	0	1	0	0	0	0	0	0
0	0	0	1	1	1	0	1	0
0	0	0	1	0	0	0	0	0
0	0	0	1	0	1	1	1	0
0	0	0	1	0	0	0	1	0
0	0	0	1	0	0	0	0	1
0	0	0	1	0	1	1	0	1
0	0	0	1	0	0	0	0	0

**Table A3:** Body sizes of lizards from Vaixell Islet included in the analysis of body growth. ID: individual identification; sex: male (M) and female (F); l_1_: SVL (mm) at the first capture; l_2_: SVL (mm) at the following recaptures; dt: time interval (in years) between recaptures (see more details in the text).

ID	Sex	l_1_	l_2_	dt
1	M	65	80	
4	M	85	85	0.25
6	M	80	85	1.75
6	M	85	86.5	1
6	M	86.5	89	1
6	M	89	89	4
7	F	78.5	80	4
8	F	68	79	2.75
8	F	79	79	2
10	F	74	78.5	1.75
10	F	78.5	79	1
10	F	79	79	1
12	M	86.5	91	4
13	F	73.5	80	3.75
18	F	73.5	75	1.75
18	F	75	76	2
18	F	76	76	2
18	F	76	76	2.25
19	F	74.5	74.5	2
19	F	74.5	75	1
19	F	75	75	1
27	M	90	90	1
30	F	62	62	1
30	F	62	75	3
30	F	75	75	2
30	F	75	76	1.25
30	F	76	76	0.83
32	M	77.5	82	1
33	M	84	87	2
34	F	71	76.5	1
34	F	76.5	78	2
36	F	71	72	1
37	F	73	76.5	2
37	F	76.5	78	4
41	F	77	77	5
44	F	73.5	75	2
44	F	75	75	2
44	F	75	79	2.08
46	F	70	73	6.08
47	F	74.5	79	5.25
48	M	83	83	1
48	M	83	84	1
48	M	84	84	3.25
49	M	82.5	82.5	3
50	M	85	88	2
50	M	88	88	2
50	M	88	90	1.25
60	M	78	79	0.83
65	M	81	84	0.83

**Table A4:** Spring and summer diet of *P. pityusensis* in Es Vaixell Islet. n = prey frequencies; n% = percentage of each prey item; np = presence frequencies; and %p = percentage of presence in faeces of each prey item.

Taxa	Spring Diet				Summer Diet			
	Frequency		Presence		Frequency		Presence	
	n	n%	np	%p	n	n%	np	%p
Gastropoda	1	0.7299	1	3.0303	2	2.0618	2	7.1428
Pseudoscorpionida	0	0	0	0	3	3.0927	2	7.1428
Araneae	2	1.4598	2	6.0606	0	0	0	0
Solifuga	1	0.7299	1	3.0303	0	0	0	0
Isopoda	5	3.6496	5	15.1515	3	3.0927	3	10.7142
Dictyoptera	1	0.7299	1	3.0303	0	0	0	0
Homoptera	4	2.9197	3	9.0909	2	2.0618	2	7.1428
Heteroptera	0	0	0	0	4	4.1237	3	10.7142
Diptera	9	6.5693	7	21.2121	19	19.5876	5	17.8571
Lepidoptera	3	2.1897	3	9.0909	5	5.1546	5	17.8571
Coleoptera	9	6.5693	7	21.2121	2	2.0618	1	3.5714
Hymenoptera	36	26.2773	7	21.2121	1	1.0309	1	3.5714
Formicidae	56	40.8759	20	60.6060	38	39.1752	10	35.7142
Arthropoda undet.	0	0	0	0	1	1.0309	1	3.5714
Larvae	4	2.9197	3	9.0909	6	6.1855	6	21.4285
Birds	3	2.1897	3	9.0909	4	4.1237	4	14.2857
Lizards	0	0	0	0	1	1.0309	1	3.5714
Carrion	1	0.7299	1	3.0303	4	4.1237	4	14.2857
Seeds	2	1.4598	2	6.0606	2	2.0618	1	3.5714
Total	140	100			97	100		
